# (*E*)-4-(Benz­yloxy)benzaldehyde thio­semicarbazone

**DOI:** 10.1107/S1600536808012671

**Published:** 2008-05-03

**Authors:** M. T. H. Tarafder, M. A. A. A. A. Islam, K. A. Crouse, Suchada Chantrapromma, Hoong-Kun Fun

**Affiliations:** a Department of Chemistry, Rajshahi University, Rajshahi 6205, Bangladesh; b Department of Chemistry, Rajshahi University of Engineering and Technology, Rajshahi 6205, Bangladesh; c Department of Chemistry, Universiti Putra Malaysia, 43400 Serdang, Selangor, Malaysia; d Department of Chemistry, Faculty of Science, Prince of Songkla University, Hat-Yai, Songkhla 90112, Thailand; e X-ray Crystallography Unit, School of Physics, Universiti Sains Malaysia, 11800 USM, Penang, Malaysia

## Abstract

In the title compound, C_15_H_15_N_3_OS, the thio­semicarbazone group adopts an *E* configuration with respect to the C=N bond. The benzaldehyde thio­semicarbazone fragment is almost planar [maximum deviation = 0.012 (1) Å], while the dihedral angle between the benz­yloxy and phenyl rings is 72.48 (5)°. In the crystal structure, mol­ecules are inter­connected by N—H⋯N and N—H⋯S hydrogen bonds, forming a two-dimensional network parallel to the *bc* plane and are further stacked along the *a* axis by π–π inter­actions [centroid–centroid separation 3.9043 (7) Å]. The crystal structure is also stabilized by C—H⋯π inter­actions.

## Related literature

For hydrogen-bond motifs, see: Bernstein *et al.* (1995 ▶). For bond-length data, see: Allen *et al.* (1987 ▶). For related structures of thio­semicarbazones, see, for example: John *et al.* (2003 ▶); Joseph *et al*. (2004 ▶). For applications and bioactivities of thio­semicarbazones, see, for example: Al-Awadi *et al.* (2008 ▶); Amoedo *et al.* (2006 ▶); Chandra *et al.*, (2001 ▶); Demertzi *et al.* (2007 ▶); Kizilcikli *et al.* (2004 ▶); Mirsha *et al.* (2006 ▶); Offiong & Martelli (1997 ▶); Sing *et al.* (2001 ▶).
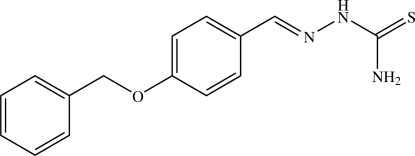


## Experimental

### 

#### Crystal data

C_15_H_15_N_3_OS
*M* *_r_* = 285.37
Monoclinic, 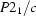

*a* = 11.0269 (1) Å
*b* = 12.6668 (2) Å
*c* = 10.8774 (1) Å
β = 116.099 (1)°
*V* = 1364.39 (3) Å^3^
*Z* = 4
Mo *K*α radiation
μ = 0.24 mm^−1^
*T* = 100.0 (1) K
0.42 × 0.31 × 0.23 mm

#### Data collection

Bruker SMART APEX2 CCD area-detector diffractometer
Absorption correction: multi-scan (*SADABS*; Bruker, 2005 ▶) *T*_min_ = 0.792, *T*_max_ = 0.947
20710 measured reflections
3983 independent reflections
3517 reflections with *I* > 2σ(*I*)
*R*_int_ = 0.027

#### Refinement

*R*[*F* ^2^ > 2σ(*F* ^2^)] = 0.035
*wR*(*F* ^2^) = 0.096
*S* = 1.03
3983 reflections
193 parameters
H atoms treated by a mixture of independent and constrained refinement
Δρ_max_ = 0.48 e Å^−3^
Δρ_min_ = −0.18 e Å^−3^

### 

Data collection: *APEX2* (Bruker, 2005 ▶); cell refinement: *APEX2* ; data reduction: *SAINT* (Bruker, 2005 ▶); program(s) used to solve structure: *SHELXTL* (Sheldrick, 2008 ▶); program(s) used to refine structure: *SHELXTL*; molecular graphics: *SHELXTL*; software used to prepare material for publication: *SHELXTL* and *PLATON* (Spek, 2003 ▶).

## Supplementary Material

Crystal structure: contains datablocks global, I. DOI: 10.1107/S1600536808012671/sj2492sup1.cif

Structure factors: contains datablocks I. DOI: 10.1107/S1600536808012671/sj2492Isup2.hkl

Additional supplementary materials:  crystallographic information; 3D view; checkCIF report

## Figures and Tables

**Table 1:** Hydrogen-bond geometry (Å, °)

*D*—H⋯*A*	*D*—H	H⋯*A*	*D*⋯*A*	*D*—H⋯*A*
N2—H1*N*2⋯S1^i^	0.880 (16)	2.467 (16)	3.3403 (10)	171.9 (14)
N3—H1*N*3⋯N1	0.895 (19)	2.229 (18)	2.6104 (16)	105.2 (13)
N3—H1*N*3⋯S1^ii^	0.895 (19)	2.815 (17)	3.5285 (11)	137.7 (14)
C10—H10*A*⋯*Cg*1^iii^	0.93	2.97	3.8325 (13)	154

Symmetry codes: (i) 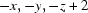; (ii) 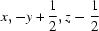; (iii) 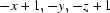. *Cg*1 is the centroid of the the C1–C6 ring.

**Fig. 1.**
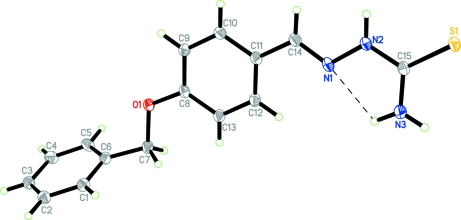
The asymmetric unit of the title compound, showing 50% probability displacement ellipsoids and the atomic numbering. The N—H···N intramolecular hydrogen bond is shown as dashed line.

**Fig. 2.**
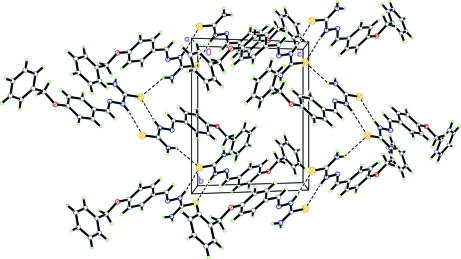
The crystal packing of the title compound, viewed along the a axis, showing two-dimensional network parallel to the bc plane. Hydrogen bonds are shown as dashed lines.

## supplementary crystallographic information

### Comment

The chemistry of thiosemicarbazones have been of immense interest because these compounds provide intriguing chelating patterns, profound biomedical properties, structural diversity and ion-sensing abilities (Al-Awadi *et al.*, 2008; Amoedo *et al.*, 2006; Demertzi *et al.*, 2007; Mirsha *et al.*, 2006; Kizilcikli *et al.*, 2004). Compounds of this type have been used as antibacterial, antifungal and antitumor agents (Sing *et al.*, 2001; Offiong *et al.*, 1997). Due to their long chain structure, they are very flexible and form linkages with a variety of metal ions (Chandra *et al.*, 2001). It was advocated that their flexibility and bioactivity arise because of the presence of the imino group (–N=CH–) in addition to thioamino moities present in the skeleton of the molecule. The title thiosemicarbazone derivative (I) was synthesized and its crystal structure is reported here. (I) is likely to have biomedical properties similar to other nitrogen-sulfur donor ligands studied by our group.

In the title compound (Fig. 1), the thiosemicarbazone adopts an *E* conformation with a *trans* configuration observed about the C═N bond. The benzaldehydethiosemicarbazone fragment is almost planar, maximum deviation 0.012 (1) Å, with the dihedral angle between the hydrazinecarbothioamide unit (S1/N1/N2/N3/C15) and the C8–C13 phenyl ring being 6.59 (5)°. The orientation of the 4-benzyloxy group is indicated by the dihedral angle between the 4-benzyloxy and the C8–C13 phenyl rings being 72.48 (5)Å and the torsion angle C8–O1–C7–C6 of 165.49 (9)°. The C15═S1 and C15—N2 bond distances are typical of a C/db S double bond and a C—N single bond, respectively. The bond lengths and angles in (I) are within normal ranges (Allen *et al.*, 1987) and show  similar trends to those of previously reported thiosemicarbazones (John *et al.*, 2003; Joseph *et al.*, 2004). An intramolecular N3-H1N3···N1 hydrogen bond forms a five-membered N3-H1N3-N1—N2—C15 ring, producing an S(5) ring motif (Bernstein *et al.*, 1995).

In the crystal packing (Fig. 2), molecules are interconnected by N—H···N and N—H···S hydrogen bonds (Table 1) into a two-dimensional network parallel to the *bc* plane and are further stacked along the *a*-axis by π···π interactions with the distances of *Cg*_1_···*Cg*_2_ = 3.9043 (7) Å: symmetry code *x*, 1/2 - *y*, -1/2 + *z*; *Cg*_1_ and *Cg*_2_ are the centroids of the C1–C6 and C8–C13 phenyl rings, respectively. The crystal also stabilized by C—H···π interactions (Table 1) involving the C1–C6 phenyl ring (centroid *Cg*_1_).

### Experimental

The title compound was synthesized by adding a solution of 4-benzyloxybenzaldehyde (2.12 g, 10 mmol) in ethanol (30 ml) to a hot solution of thiosemicarbazide (0.91 g, 10 mmol) in ethanol (100 ml). The mixture was refluxed for 2 hrs and subsequently cooled to room temperature. The light yellow precipitate of the title compound was separated by filtration, washed with ethanol and dried *in vacuo* over anhydrous CaCl_2_. (Yield: 1.75 g, 61%), and was then dissolved in chloroform (0.11 g in 50 ml) and allowed to stand at room temperature (288–293 K) for 20 days. Yellow single crystals of the title compound were obtained after recrystallization from a solution of chloroform/toluene (30:7 *v*/*v*) after 12 days at room temperature, *M*.p 446 K.

### Refinement

H atoms bound to N atoms were located from a difference Fourier map and refined freely with isotropic displacement parameters. The remaining H atoms were positioned geometrically and allowed to ride on their parent atoms, with d(C—H) = 0.93 Å, for aromatic, 0.97 Å, for CH_2_ and *U*_iso_ = 1.2*U*_eq_(C). The highest residual electron density peak is located at 0.69 Å from C8 and the deepest hole is located at 1.19 Å from C12.

### Crystal data

**Table:**

C_15_H_15_N_3_OS	*F*_000_ = 600
*M**_r_* = 285.37	*D*_x_ = 1.389 Mg m^−^^3^
Monoclinic, *P*2_1_/*c*	Melting point: 446 K
Hall symbol: -P 2ybc	Mo *K*α radiation λ = 0.71073 Å
*a* = 11.0269 (1) Å	Cell parameters from 3983 reflections
*b* = 12.6668 (2) Å	θ = 2.1–30.0º
*c* = 10.8774 (1) Å	µ = 0.24 mm^−^^1^
β = 116.099 (1)º	*T* = 100.0 (1) K
*V* = 1364.39 (3) Å^3^	Block, colorless
*Z* = 4	0.42 × 0.31 × 0.23 mm

### Data collection

**Table:**

Bruker SMART APEX2 CCD area-detector diffractometer	3983 independent reflections
Radiation source: fine-focus sealed tube	3517 reflections with *I* > 2σ(*I*)
Monochromator: graphite	*R*_int_ = 0.027
Detector resolution: 8.33 pixels mm^-1^	θ_max_ = 30.0º
*T* = 100.0(1) K	θ_min_ = 2.1º
ω scans	*h* = −15→14
Absorption correction: multi-scan(SADABS; Bruker, 2005)	*k* = −17→17
*T*_min_ = 0.792, *T*_max_ = 0.947	*l* = −15→14
20710 measured reflections	

### Refinement

**Table:**

Refinement on *F*^2^	Secondary atom site location: difference Fourier map
Least-squares matrix: full	Hydrogen site location: inferred from neighbouring sites
*R*[*F*^2^ > 2σ(*F*^2^)] = 0.035	H atoms treated by a mixture of independent and constrained refinement
*wR*(*F*^2^) = 0.096	*w* = 1/[σ^2^(*F*_o_^2^) + (0.0529*P*)^2^ + 0.4873*P*] where *P* = (*F*_o_^2^ + 2*F*_c_^2^)/3
*S* = 1.04	(Δ/σ)_max_ = 0.001
3983 reflections	Δρ_max_ = 0.48 e Å^−^^3^
193 parameters	Δρ_min_ = −0.18 e Å^−^^3^
Primary atom site location: structure-invariant direct methods	Extinction correction: none

### Special details

**Table:**

Experimental. The low-temperature data was collected with the Oxford Cyrosystem Cobra low-temperature attachment.
Geometry. All e.s.d.'s (except the e.s.d. in the dihedral angle between two l.s. planes) are estimated using the full covariance matrix. The cell e.s.d.'s are taken into account individually in the estimation of e.s.d.'s in distances, angles and torsion angles; correlations between e.s.d.'s in cell parameters are only used when they are defined by crystal symmetry. An approximate (isotropic) treatment of cell e.s.d.'s is used for estimating e.s.d.'s involving l.s. planes.
Refinement. Refinement of *F*^2^ against ALL reflections. The weighted *R*-factor *wR* and goodness of fit *S* are based on *F*^2^, conventional *R*-factors *R* are based on *F*, with *F* set to zero for negative *F*^2^. The threshold expression of *F*^2^ > σ(*F*^2^) is used only for calculating *R*-factors(gt) *etc*. and is not relevant to the choice of reflections for refinement. *R*-factors based on *F*^2^ are statistically about twice as large as those based on *F*, and *R*- factors based on ALL data will be even larger.

### Fractional atomic coordinates and isotropic or equivalent isotropic displacement parameters (Å^2^)

**Table:**

	*x*	*y*	*z*	*U*_iso_*/*U*_eq_	
S1	−0.13297 (3)	0.13427 (2)	0.97645 (3)	0.01819 (8)	
O1	0.32553 (8)	0.07638 (6)	0.33345 (8)	0.01820 (17)	
N1	0.04289 (10)	0.10009 (8)	0.73443 (9)	0.01734 (18)	
N2	0.00675 (10)	0.08433 (8)	0.83961 (9)	0.01797 (19)	
N3	−0.13647 (10)	0.22436 (8)	0.75438 (10)	0.01930 (19)	
C1	0.44624 (11)	0.26954 (9)	0.20175 (11)	0.0185 (2)	
H1A	0.4399	0.3177	0.2632	0.022*	
C2	0.53207 (12)	0.29079 (9)	0.14214 (11)	0.0190 (2)	
H2A	0.5835	0.3522	0.1645	0.023*	
C3	0.54055 (12)	0.21970 (9)	0.04895 (11)	0.0196 (2)	
H3A	0.5973	0.2334	0.0081	0.024*	
C4	0.46359 (13)	0.12808 (9)	0.01727 (12)	0.0214 (2)	
H4A	0.4685	0.0808	−0.0458	0.026*	
C5	0.37939 (12)	0.10629 (9)	0.07858 (11)	0.0206 (2)	
H5A	0.3295	0.0441	0.0577	0.025*	
C6	0.36955 (11)	0.17766 (9)	0.17140 (11)	0.0174 (2)	
C7	0.27374 (12)	0.16007 (10)	0.23367 (11)	0.0203 (2)	
H7A	0.2647	0.2244	0.2774	0.024*	
H7B	0.1853	0.1410	0.1630	0.024*	
C8	0.26799 (11)	0.06651 (8)	0.42162 (10)	0.0158 (2)	
C9	0.32639 (11)	−0.00843 (9)	0.52540 (11)	0.0178 (2)	
H9A	0.3968	−0.0507	0.5288	0.021*	
C10	0.27868 (12)	−0.01940 (9)	0.62342 (11)	0.0177 (2)	
H10A	0.3173	−0.0696	0.6922	0.021*	
C11	0.17333 (11)	0.04386 (8)	0.62022 (10)	0.0160 (2)	
C12	0.11361 (11)	0.11615 (9)	0.51313 (11)	0.0168 (2)	
H12A	0.0419	0.1574	0.5084	0.020*	
C13	0.15942 (11)	0.12737 (9)	0.41392 (11)	0.0170 (2)	
H13A	0.1181	0.1752	0.3426	0.020*	
C14	0.12869 (11)	0.03475 (9)	0.72786 (11)	0.0175 (2)	
H14A	0.1629	−0.0190	0.7923	0.021*	
C15	−0.08583 (11)	0.14858 (8)	0.84847 (11)	0.0156 (2)	
H1N2	0.0392 (16)	0.0301 (13)	0.8951 (16)	0.025 (4)*	
H1N3	−0.1132 (17)	0.2256 (13)	0.6853 (18)	0.031 (4)*	
H2N3	−0.1995 (18)	0.2624 (14)	0.7562 (17)	0.030 (4)*	

### Atomic displacement parameters (Å^2^)

**Table:**

	*U*^11^	*U*^22^	*U*^33^	*U*^12^	*U*^13^	*U*^23^
S1	0.02249 (15)	0.01892 (14)	0.01894 (14)	0.00132 (10)	0.01441 (11)	0.00114 (9)
O1	0.0202 (4)	0.0215 (4)	0.0181 (3)	0.0037 (3)	0.0132 (3)	0.0039 (3)
N1	0.0192 (5)	0.0209 (4)	0.0157 (4)	−0.0012 (3)	0.0111 (4)	0.0006 (3)
N2	0.0217 (5)	0.0200 (4)	0.0176 (4)	0.0032 (4)	0.0136 (4)	0.0035 (3)
N3	0.0189 (5)	0.0224 (5)	0.0197 (4)	0.0039 (4)	0.0114 (4)	0.0045 (4)
C1	0.0198 (5)	0.0211 (5)	0.0152 (4)	0.0028 (4)	0.0082 (4)	0.0004 (4)
C2	0.0184 (5)	0.0208 (5)	0.0170 (5)	−0.0012 (4)	0.0070 (4)	0.0012 (4)
C3	0.0204 (5)	0.0239 (5)	0.0176 (5)	0.0023 (4)	0.0112 (4)	0.0042 (4)
C4	0.0292 (6)	0.0210 (5)	0.0186 (5)	0.0021 (4)	0.0147 (5)	0.0004 (4)
C5	0.0253 (6)	0.0196 (5)	0.0191 (5)	−0.0028 (4)	0.0118 (4)	0.0004 (4)
C6	0.0167 (5)	0.0224 (5)	0.0144 (4)	0.0029 (4)	0.0079 (4)	0.0038 (4)
C7	0.0182 (5)	0.0272 (6)	0.0185 (5)	0.0041 (4)	0.0107 (4)	0.0064 (4)
C8	0.0163 (5)	0.0182 (5)	0.0155 (4)	−0.0017 (4)	0.0095 (4)	−0.0013 (4)
C9	0.0194 (5)	0.0174 (5)	0.0202 (5)	0.0021 (4)	0.0121 (4)	0.0007 (4)
C10	0.0205 (5)	0.0170 (5)	0.0185 (5)	0.0014 (4)	0.0111 (4)	0.0021 (4)
C11	0.0177 (5)	0.0170 (5)	0.0158 (4)	−0.0014 (4)	0.0097 (4)	−0.0011 (4)
C12	0.0151 (5)	0.0206 (5)	0.0163 (5)	0.0010 (4)	0.0083 (4)	0.0001 (4)
C13	0.0157 (5)	0.0210 (5)	0.0152 (4)	0.0017 (4)	0.0077 (4)	0.0021 (4)
C14	0.0200 (5)	0.0181 (5)	0.0171 (4)	−0.0004 (4)	0.0106 (4)	0.0011 (4)
C15	0.0146 (5)	0.0173 (5)	0.0160 (4)	−0.0024 (4)	0.0078 (4)	−0.0010 (4)

### Geometric parameters (Å, °)

**Table:**

S1—C15	1.6964 (11)	C4—H4A	0.9300
O1—C8	1.3688 (12)	C5—C6	1.3946 (15)
O1—C7	1.4430 (13)	C5—H5A	0.9300
N1—C14	1.2826 (14)	C6—C7	1.5014 (15)
N1—N2	1.3815 (12)	C7—H7A	0.9700
N2—C15	1.3417 (14)	C7—H7B	0.9700
N2—H1N2	0.880 (17)	C8—C13	1.3950 (15)
N3—C15	1.3335 (14)	C8—C9	1.3966 (15)
N3—H1N3	0.894 (17)	C9—C10	1.3879 (14)
N3—H2N3	0.853 (18)	C9—H9A	0.9300
C1—C2	1.3886 (15)	C10—C11	1.3990 (15)
C1—C6	1.3903 (16)	C10—H10A	0.9300
C1—H1A	0.9300	C11—C12	1.3979 (15)
C2—C3	1.3894 (15)	C11—C14	1.4606 (14)
C2—H2A	0.9300	C12—C13	1.3856 (14)
C3—C4	1.3887 (16)	C12—H12A	0.9300
C3—H3A	0.9300	C13—H13A	0.9300
C4—C5	1.3884 (16)	C14—H14A	0.9300
			
C8—O1—C7	116.23 (8)	C6—C7—H7A	109.9
C14—N1—N2	116.20 (9)	O1—C7—H7B	109.9
C15—N2—N1	118.53 (9)	C6—C7—H7B	109.9
C15—N2—H1N2	121.1 (10)	H7A—C7—H7B	108.3
N1—N2—H1N2	120.2 (10)	O1—C8—C13	123.89 (9)
C15—N3—H1N3	118.6 (11)	O1—C8—C9	115.94 (9)
C15—N3—H2N3	117.4 (11)	C13—C8—C9	120.16 (9)
H1N3—N3—H2N3	123.1 (15)	C10—C9—C8	119.55 (10)
C2—C1—C6	121.31 (10)	C10—C9—H9A	120.2
C2—C1—H1A	119.3	C8—C9—H9A	120.2
C6—C1—H1A	119.3	C9—C10—C11	120.98 (10)
C1—C2—C3	119.57 (11)	C9—C10—H10A	119.5
C1—C2—H2A	120.2	C11—C10—H10A	119.5
C3—C2—H2A	120.2	C12—C11—C10	118.52 (9)
C4—C3—C2	119.52 (10)	C12—C11—C14	121.22 (10)
C4—C3—H3A	120.2	C10—C11—C14	120.25 (10)
C2—C3—H3A	120.2	C13—C12—C11	121.11 (10)
C5—C4—C3	120.78 (10)	C13—C12—H12A	119.4
C5—C4—H4A	119.6	C11—C12—H12A	119.4
C3—C4—H4A	119.6	C12—C13—C8	119.60 (10)
C4—C5—C6	120.02 (11)	C12—C13—H13A	120.2
C4—C5—H5A	120.0	C8—C13—H13A	120.2
C6—C5—H5A	120.0	N1—C14—C11	120.71 (10)
C1—C6—C5	118.79 (10)	N1—C14—H14A	119.6
C1—C6—C7	119.49 (10)	C11—C14—H14A	119.6
C5—C6—C7	121.65 (10)	N3—C15—N2	117.16 (9)
O1—C7—C6	108.91 (9)	N3—C15—S1	122.05 (8)
O1—C7—H7A	109.9	N2—C15—S1	120.78 (8)
			
C14—N1—N2—C15	−177.98 (10)	C13—C8—C9—C10	−2.17 (16)
C6—C1—C2—C3	0.83 (17)	C8—C9—C10—C11	−0.31 (17)
C1—C2—C3—C4	−0.35 (17)	C9—C10—C11—C12	2.21 (16)
C2—C3—C4—C5	−0.62 (18)	C9—C10—C11—C14	−177.07 (10)
C3—C4—C5—C6	1.13 (18)	C10—C11—C12—C13	−1.66 (16)
C2—C1—C6—C5	−0.32 (16)	C14—C11—C12—C13	177.61 (10)
C2—C1—C6—C7	−177.44 (10)	C11—C12—C13—C8	−0.77 (17)
C4—C5—C6—C1	−0.65 (17)	O1—C8—C13—C12	−175.94 (10)
C4—C5—C6—C7	176.40 (11)	C9—C8—C13—C12	2.71 (16)
C8—O1—C7—C6	165.49 (9)	N2—N1—C14—C11	179.67 (9)
C1—C6—C7—O1	−109.78 (11)	C12—C11—C14—N1	−7.05 (17)
C5—C6—C7—O1	73.19 (13)	C10—C11—C14—N1	172.21 (10)
C7—O1—C8—C13	4.11 (15)	N1—N2—C15—N3	−0.34 (15)
C7—O1—C8—C9	−174.59 (10)	N1—N2—C15—S1	−179.21 (8)
O1—C8—C9—C10	176.58 (10)		

### Hydrogen-bond geometry (Å, °)

**Table:**

*D*—H···*A*	*D*—H	H···*A*	*D*···*A*	*D*—H···*A*
N2—H1N2···S1^i^	0.880 (16)	2.467 (16)	3.3403 (10)	171.9 (14)
N3—H1N3···N1	0.895 (19)	2.229 (18)	2.6104 (16)	105.2 (13)
N3—H1N3···S1^ii^	0.895 (19)	2.815 (17)	3.5285 (11)	137.7 (14)
C10—H10A···Cg1^iii^	0.93	2.97	3.8325 (13)	154

Symmetry codes: (i) −*x*, −*y*, −*z*+2;  (ii) *x*, −*y*+1/2, *z*−1/2; (iii) −*x*+1, −*y*, −*z*+1.

## References

[bb1] Al-Awadi, N. A., Shuaib, N. A., Abbas, A., EI-Sherif, A. A., EI-Dissouky, A. & Al-Saleh, E. (2008). *Bioinorg. Chem. Appl* doi:10.1155/2008/479897. 10.1155/2008/479897 PMC2268950 18364993

[bb2] Allen, F. H., Kennard, O., Watson, D. G., Brammer, L., Orpen, A. G. & Taylor, R. (1987). *J. Chem. Soc. Perkin Trans. 2*, pp. S1–S19.

[bb3] Amoedo, A., Adrio, L. A., Antelo, J. M., Martinez, J., Pereira, M. T., Fernandez, A. & Vila, J. M. (2006). *Eur. J. Inorg. Chem.* pp. 3016–3021.

[bb4] Bernstein, J., Davis, R. E., Shimoni, L. & Chang, N.-L. (1995). *Angew. Chem. Int. Ed. Engl.* **34**, 1555–1573.

[bb5] Bruker (2005). *APEX2*, *SAINT* and *SADABS* Bruker AXS Inc., Madison, Wisconsin, USA.

[bb6] Chandra, S., Sangeetika & Rathi, A. (2001). *J. Saudi Chem. Soc.* **5**, 175–182.

[bb7] Demertzi, D. K., Varadinova, T., Genova, P., Souza, P. & Demertzi, M. A. (2007). *Bioinorg. Chem. Appl.* doi:10.1155/2007/56165. 10.1155/2007/56165 PMC1876625 17541481

[bb8] John, R. P., Sreekanth, A., Kurup, M. R. P., Usman, A., Razak, I. A. & Fun, H. K. (2003). *Spectrochim. Acta A*, **59**, 1349–1358. 10.1016/s1386-1425(02)00332-3 12659904

[bb9] Joseph, M., Suni, V., Nayar, C. R., Kurup, M. R. P. & Fun, H. K. (2004). *J. Mol. Struct.* **705**, 63–70.

[bb10] Kizilcikli, I., Ulkuseven, B., Dasdemir, Y. & Akkurt, B. (2004). *Synth. React. Inorg. Met.-Org. Chem.*, **34**, 653–665.

[bb11] Mirsha, D., Nasker, S., Drew, M. G. B. & Chattopadhay, S. K. (2006). *Inorg. Chim. Acta*, **359**, 585–592.

[bb12] Offiong, O. E. & Martelli, S. (1997). *Transition Met. Chem.* **22**, 263–269.

[bb13] Sheldrick, G. M. (2008). *Acta Cryst.* A**64**, 112–122. 10.1107/S0108767307043930 18156677

[bb14] Sing, N. K., Sing, S. B., Shrivastav, A. & Sing, S. M. (2001). *Proc. Indian Acad. Sci. Chem. Sess* **113**, 257–273.

[bb15] Spek, A. L. (2003). *J. Appl. Cryst.* **36**, 7–13.

